# Anti-proliferative effects, cell cycle G_2_/M phase arrest and blocking of chromosome segregation by probimane and MST-16 in human tumor cell lines

**DOI:** 10.1186/1471-2210-5-11

**Published:** 2005-06-20

**Authors:** Da Yong Lu, Min Huang, Cheng Hui Xu, Wei Yi Yang, Chao Xin Hu, Li Ping Lin, Lin Jiang Tong, Mei Hong Li, Wei Lu, Xiong Wen Zhang, Jian Ding

**Affiliations:** 1 School of Life Sciences, Shanghai University; Shanghai 200436, PR China; 2 Division of Anti-tumor Pharmacology, Shanghai Institute of Materia Medica, Shanghai Institutes for Biological Sciences, Chinese Academy of Sciences, Shanghai 201203, PR China; 3 Graduate School of Chinese Academy of Sciences, Shanghai 200031, PR China

## Abstract

**Background:**

Anticancer bisdioxopiperazines, including ICRF-154, razoxane (Raz, ICRF-159) and ICRF-193, are a family of anticancer agents developed in the UK, especially targeting metastases of neoplasms. Two other bisdioxopiperazine derivatives, probimane (Pro) and MST-16, were synthesized at the Shanghai Institute of Materia Medica, Chinese Academy of Sciences, Shanghai, China. Cytotoxic activities and mechanisms of Raz (+)-steroisomer (ICRF-187, dexrazoxane), Pro and MST-16 against tumor cells were evaluated by MTT colorimetry, flow cytometry and karyotyping.

**Results:**

Pro was cytotoxic to human tumor cell lines *in vitro *(IC_50_<50 μM for 48 h). Four human tumor cell lines (SCG-7901, K562, A549 and HL60) were susceptible to Pro at low inhibitory concentrations (IC_50 _values < 10 μM for 48 h). Although the IC_50 _against HeLa cell line of vincristine (VCR, 4.56 μM), doxorubicin (Dox, 1.12 μM) and 5-fluoruouracil (5-Fu, 0.232 μM) are lower than Pro (5.12 μM), ICRF-187 (129 μM) and MST-16 (26.4 μM), VCR, Dox and 5-Fu shows a low dose-related – high cytotoxic activity. Time-response studies showed that the cytotoxic effects of Pro are increased for 3 days in human tumor cells, whereas VCR, Dox and 5-Fu showed decreased cytotoxic action after 24 h. Cell cycle G_2_/M phase arrest and chromosome segregation blocking by Pro and MST-16 were noted. Although there was similar effects of Pro and MST-16 on chromosome segregation blocking action and cell cycle G_2_/M phase arrest at 1- 4 μM, cytotoxicity of Pro against tumor cells was higher than that of MST-16 *in vitro *by a factor of 3- 10 folds. Our data show that Pro may be more effective against lung cancer and leukemia while ICRF-187 and MST-16 shows similar IC_50 _values only against leukemia.

**Conclusion:**

It suggests that Pro has a wider spectrum of cytotoxic effects against human tumor cells than other bisdioxopiperazines, especially against solid tumors, and with a single cytotoxic pathway of Pro and MST-16 affecting chromosome segregation and leading also to cell G_2_/ M phase arrests, which finally reduces cell division rates. Pro may be more potent than MST-16 in cytotoxicity. High dose- and time- responses of Pro, when compared with VCR, 5-Fu and Dox, were seen that suggest a selectivity of Pro against tumor growth. Compounds of bisdioxopiperazines family may keep up their cytotoxic effects longer than many other anticancer drugs.

## Background

**Figure 1:**
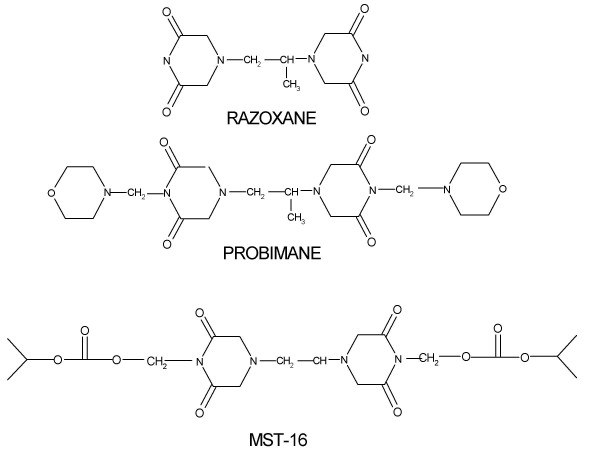
Structural formulae of three bisdioxopiperazines

Bisdioxopiperazines, including ICRF-154, razoxane (ICRF-159, *Raz*); ICRF-186 and ICRF-187), two stereo-isomers of Raz, and ICRF-193, developed in the UK, were some of the earliest agents found against a murine spontaneous metastatic model (Lewis lung carcinoma) in 1969 [1]. Many papers and projects have dealt with their potential use and mechanisms since that time. Three main mechanisms of bisdioxopiperazine action have been investigated, including assisting in radiotherapy, [2,3] overcoming multi-drug resistance (MDR) of daunorubicin and doxorubicin to leukemias [4,5] and inhibiting topoisomerase II [6,7]. More importantly, Raz has been licensed for cardioprotectant of anticancer anthrocyclines in more countries. Since bisdioxopiperazines represents a unique family of antimetastatic agents that are structurally conservative in their pharmacological actions, two new derivatives, probimane [1,2-bis (N^4^-morpholine-3, 5-dioxopeprazine-1-yl) propane; AT-2153, Pro] and MST-16, 1, 2- bis (4- isobutoxycarbonyloxymethyl- 3, 5- dioxopiperazin-1- yl) ethane were synthesized at this institute in Shanghai, China. [8,9]. Apart from data of anti-tumor activity [10-12], the pharmacological mechanisms of Pro as Raz, like the detoxication of *Adriamycin *(*ADR*), induced cardiotoxicities and synergism with *ADR *against leukemias were reported at Henan Academy of Medicine, Henan, China [13]. As the main researchers of Pro, we reported some novel biological actions of Pro, including the inhibition of the activity of calmodulin (*CaM*), a cell-signal regulator, which can explain anticancer actions and the combined cytotoxic effect of *Pro *with *ADR *[13,14] inhibiting lipoperoxidation (*LPO*) of erythrocytes [15], down-regulating sialic acid synthesis in tumors [16] and blocking the binding of fibrinogen to leukemia cells [17]. MST-16, as a licensed drug in Japan since 1994, was permitted for direct use in leukemia chemotherapy, mainly against adult T-cell leukemia treatment [18]. Structural formulae of the three bisdioxopiperazines are represented in Figure 1.

As a new bisdioxopiperazine, the pharmacological characters and features of Pro are intriguing. Increased understanding of the advantages and disadvantages of the two compounds is a first step for promoting applications of Pro and MST-16. Therefore, in depth pharmacological evaluation was carried out. Tumors studied are from 7 different organs of origin – two gastric tumor cell line (SCG-7901, MKN-28), a lung tumor cell line (A549), a colon cancer cell line (HCT-116), two mammary tumor cell lines (MDA-MB-435, MDA-MB-468), one hepatic tumor cell line (BEL7402), two leukemia cell line (HL-60 and K562) and an uteric cervical tumor cell line (HeLa). In addition, time- and concentration-dependent relations to classify the effectiveness of different therapeutic schedules and schemes of Pro and MST-16 therapy have been addressed.

## Results

### Cytotoxic effects of Pro and MST-16 against human tumor cell lines

**Figure 2:**
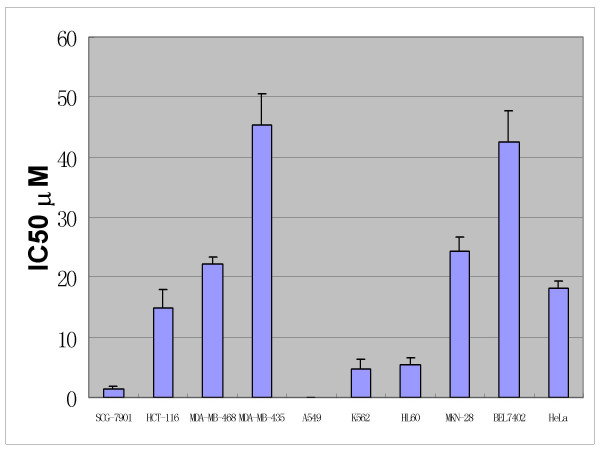
Anticancer activities of probimane *in vitro*. MTT method is used. Pro exposures for 48 h at 5 different concentrations. n = 3 and in 2 independent tests.

Data on the anticancer effects of Pro using 10 human tumor cell lines *in vitro *are showed in Figure 2 and Table 1. Pro had anticancer effects *in vitro *at clinical acceptable concentrations (IC_50 _values < 50 μM) by MTT methods. The IC_50 _values of Pro are 1.3672 ± 0.6230 μM, 24.314 ± 5.465 μM, 14.476 ± 3.085 μM, 45.325 ± 5.335, 22.169 ± 1.250, 0.02947 ± 0.02456 μM, 5.3417 ± 1.245 μM, 4.786 ± 1.556, 42.457 ± 2.325 μM and 18.238 ± 1.112 μM representing tumor cells of SCG-7901 and MKN- 28 (two human gastric tumor cell lines), HCT-116 (a human colon tumor cell line), MDA-MB-435 and MDA-MB-468 (two human mammary tumor cell lines), A549 (a human lung tumor cell line) and HL60 and K562 (two human leukemia cell lines), BEL-7402 (a human hepatic tumor cell line) and HeLa cell (a human uteric cervical tumor cell line) respectively (Figure 2). Among these tumor cell lines, Pro is more effective to SCG-7901 (a gastric cancer cell line), A549 (a lung cancer cell line) and HL60 and K562 (two leukemia cell lines), the IC_50 _values being ≤10 μM.

**Table 1:** The IC_50 _values of Pro in different human tumor cell lines for 48 h. MTT method was used.

Cell origin	Cell types	IC_50 _μM; mean ± SD
Gastric	SCG-7901	1.3672 ± 0.6230
	MKN-28	24.314 ± 5.465
Colon	HCT-116	14.476 ± 3.085
Mammary	MDA-MB-435	45.325 ± 5.335
	MDA-MB-468	22.169 ± 1.250
Pulmonary	A549	0.02947 ± 0.02456
Leukemia	HL-60	5.3417 ± 1.245
	K562	4.786 ± 1.556
Uteric cervical	HeLa	18.238 ± 1.112
Hepatic	BEL-7402	42.457 ± 2.325

### Comparison of the cytotoxic effects of bisdioxopiperazines with other drugs

The cytotoxic effects against tumor cell lines (p388, HL-60 and HeLa cells) are included in Table 1. Although IC_50_s of Dox, VCR and 5-Fu are lower than that of Pro, the greatest inhibitory rates of Pro at high concentrations are seen (Table 2). No inhibitory difference between low and high concentrations of Dox, VCR and 5-Fu was observed (Table 3). Generally, the LD_50 _of VCR and Dox in experimental animals and humans are dramatically lower than Pro. These results suggest more difficult management and wider toxicities of these drugs in their application in the clinics, suggesting Pro may avoid these drawbacks.

**Table 2:** Cytotoxic effects of anticancer drugs against tumor cell lines *in vitro*; drug exposure for 48 h

Compounds	IC_50 _μM		
	
	P388	HL-60	HeLa
Doxorubicin	11.7	0.005	1.12
Vincristine	No effect	0.05	4.56
5-fluorouracil	22.6	0.04	0.23
Probimane	64.6	1.97	5.12
ICRF-187	64.0	3.73	129
MST-16	5.23	33.4	26.4

**Table 3:** Dose- response relations between anti-cancer drugs for cytotoxic effect against human leukemia cell line HL-60 for 24 h; * P < 0.01; n = 3;

Compounds	Concentrations	OD values Mean ± SD	Percentage inhibition %
Control	--	1.229 ± 0.125	--
Probimane	10.0	0.298 ± 0.010*	75.6
	2.0	0.260 ± 0.005*	78.9
	0.4	1.142 ± 0.010	7.1
	0.08	1.199 ± 0.012	2.4
Doxorubicin	10.0	0.256 ± 0.021*	79.2
	2.0	0.266 ± 0.013*	78.3
	0.4	0.312 ± 0.016*	74.5
	0.08	0.408 ± 0.031*	66.9
5-Fluorouracil	5.0	0.421 ± 0.021*	65.6
	1.0	0.518 ± 0.012*	57.9
	0.2	0.585 ± 0.025*	54.4
	0.04	0.892 ± 0.038*	27.5
Vincristine	5.0	0.425 ± 0.010*	65.4
	1.0	0.423 ± 0.009*	65.6
	0.2	0.401 ± 0.009*	67.4
	0.04	0.394 ± 0.012*	68.0

### Comparison of anti-tumor effects of probimane and MST-16 and their time- response relationships

**Figure 3:**
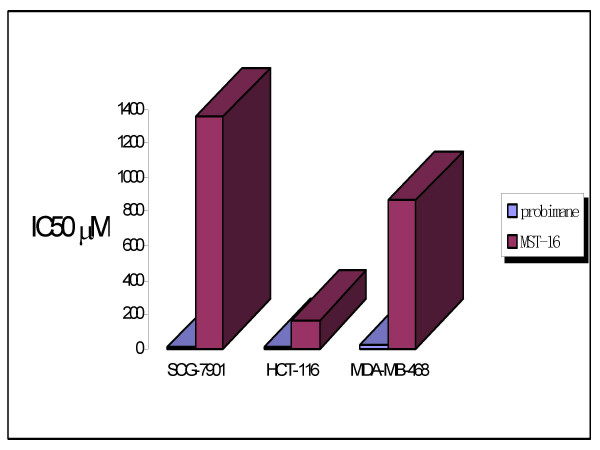
The IC_50 _of probimane and MST-16 on 3 human tumor cell lines (SCG-7901, HCT-116 and MDA-MB-468) for 48 h exposure. MTT method was used.

Cytotoxic effects (IC_50_) of probimane and MST-16 against tumor cells were compared (Figure 3).

**Figure 4:**
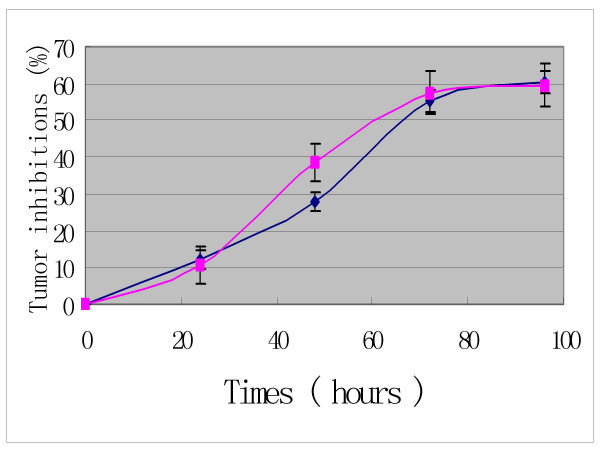
Time – response curve of probimane inhibiting a human mammary cell line (MDA-MB-468). MTT method was used. A: Pro concentration at 5 μM (dark); B: Pro concentration at 0.5 μM (purple).

**Figure 5:**
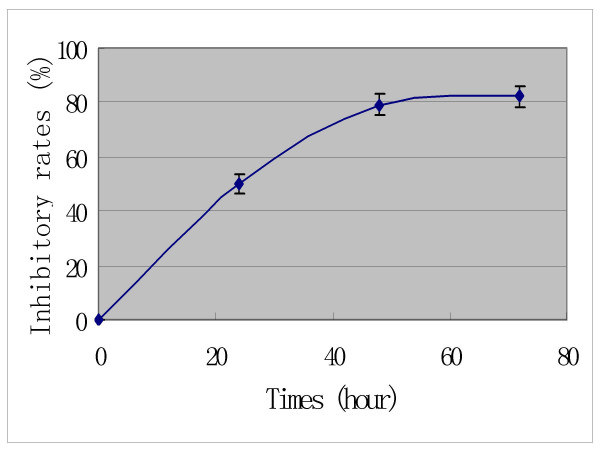
Time- response curve of probimane inhibiting a human gastric tumor cell line SCG-7901. MTT method was used. A: Pro concentration at 5 μM

**Figure 6:**
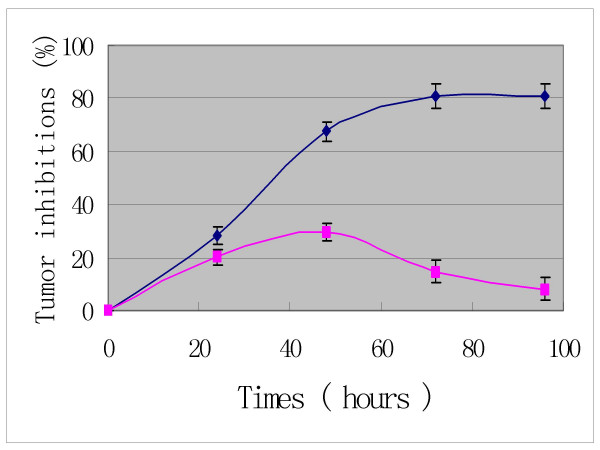
Time- response curve of probimane inhibiting a human mammary cell line (MDA-MB-435). MTT method was used. A: Pro concentration at 50 μM (dark); B: Pro concentration at 5 μM (purple).

**Figure 7:**
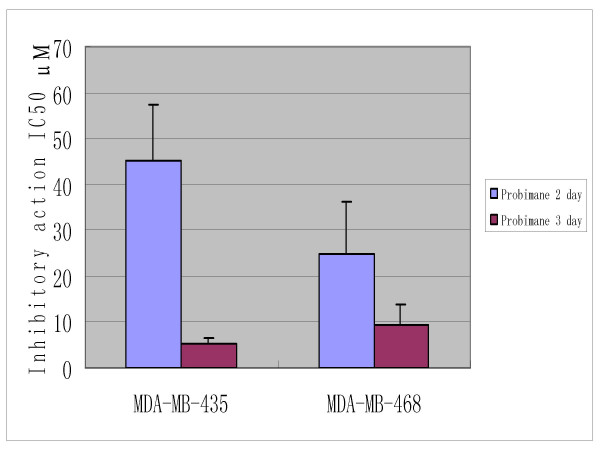
Differences of anticancer effects (IC_50_) of probimane for different exposure intervals by a MTT method, n = 3 for 2 independent tests.

**Figure 8:**
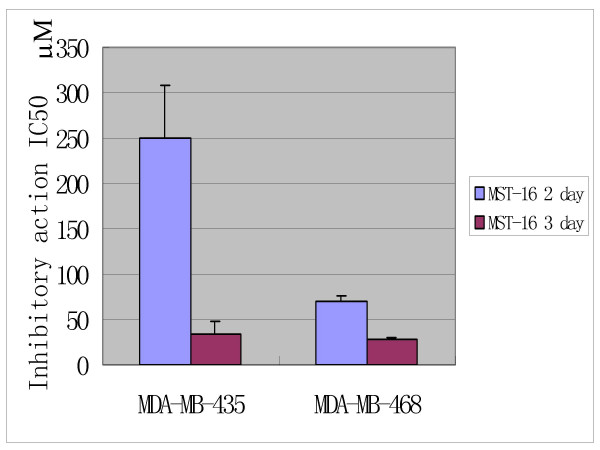
Differences of anticancer effects (IC_50_) of MST-16 for different exposure intervals by a MTT method, n = 3 for 2 independent tests.

In addition, the time- response curves indicate that the anti-tumor effects of Pro increase to a climax over 3 days of drug exposure (Figures 4, 5 and 6). The cytotoxic effects of Pro persist or rise with time, whereas those of VCR, Dox and 5-Fu decrease after 24 h (Table 4). IC_50 _of both Pro and MST-16 reduces dramatically by 72 h from 48 h. (Figures 7 and 8). The reductions of IC_50 _for both agents Pro or MST-16 depend on the cell line. IC_50 _ratios of Pro and MST-16 for 3 days relative to 2 days against the most metastatic phenotype tumor cell line MDA-MB-435, are 8.9 and 7.5 times higher, and 2.6 times for the medium metastatic phenotype of MDA-MB-468 cells.

**Table 4:** The time- response relations between different anticancer drugs for cyto-toxic effects against leukemia cell line HL-60; N = 3, probimane, Pro; 5-fluorouracil, 5-Fu; doxorubicin, Dox; vincristine, VCR; ICRF-187, (+) stereo-isomer of razoxane

Compounds	Concentrations	Percentage inhibition %		
		
	μM	24 h	48 h	72 h
Pro	10	75.6	78.5	75.9
5-Fu	2	65.8	53.5	51.1
Dox	4	78.3	72.4	72.3
VCR	2	65.4	59.0	57.1
ICRF-187	10	47.6	37.8	42.9
MST-16	10	47.8	5.6	0.0

### G_2 _and M phase arrests induced by Pro or MST-16

**Figure 9:**
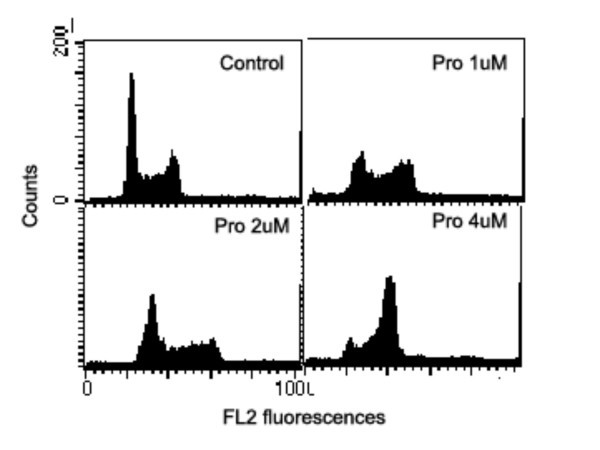
G_2_/M phase arrests of human mammary tumor cell line (MDA-MB-435 cell) exposed to probimane at different concentrations for 20 h.

**Figure 10:**
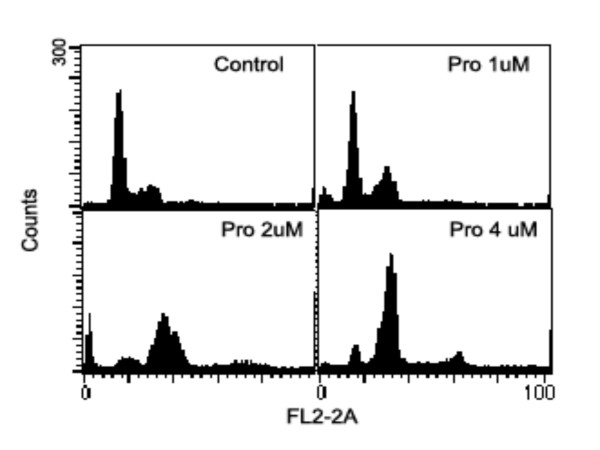
G_2_/M phase arrests of a human colon tumor cell line (HCT-116 cell) exposed to probimane at different concentrations for 20 h.

**Figure 11:**
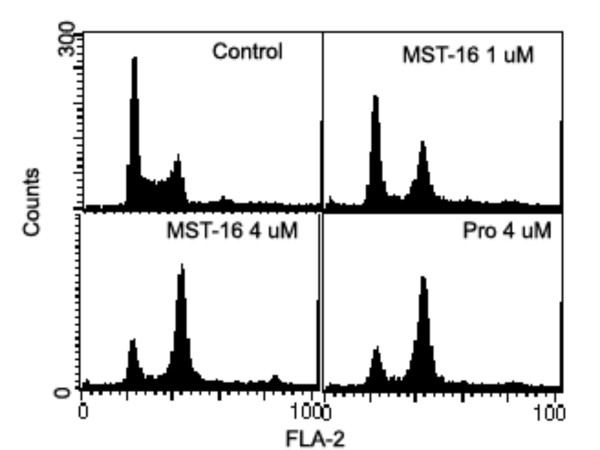
G_2_/M phase arrests of human mammary tumor cell line (MDA-MB-435 cell) exposed to MST-16 and probimane for 20 h. A: vehicle control; B: MST-16 0.8 μM; C: MST-16 4.0 μM; D: Pro 2.0 μM

**Figure 12:**
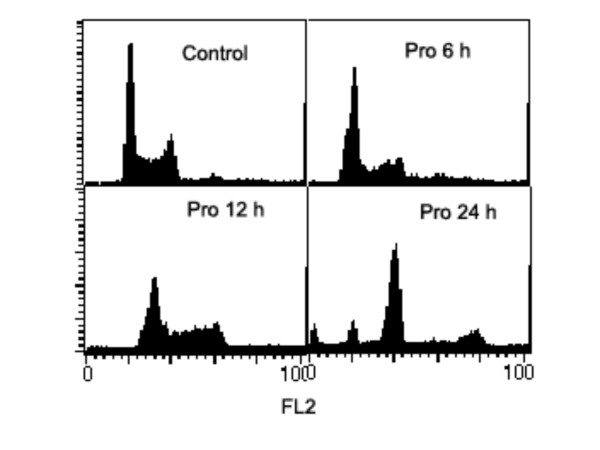
G_2_/M phase arrests of human mammary tumor cell line (MDA-MB-435 cell) exposed to probimane for different times. A: vehicle control; B: Pro 6 h; C: Pro 12 h; D: Pro 24 h

Our data shows that both probimane (Pro) and MST-16 can arrest tumor cells in G_2 _and M phases of the cell cycle. Dose- and/ or concentration- dependency are observed in G_2 _and M arrests (Figures 9 to 12), and the arresting effect of Pro on MDA-MB-435 and HCT-116 is only 2 times higher for MST-16 at equivalent concentrations. Pro at 4 μM can increase G_2_/M accumulation from 16.8 % (vehicle control) to 86.4 % after 24 h (p < 0.001, n = 3).

### Chromosome segregation inhibition by Pro and MST-16

**Figure 13:**
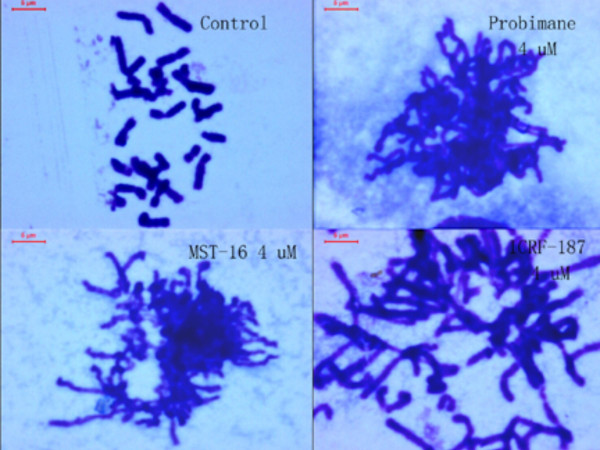
Chromosomal behaviors of human mammary tumor cell line- MDA-MB-435 incubated with bisdioxopiperazines. A: control; B: probimane 4 μM; C: MST-16 4μM; D: ICRF-187 4μM.

**Figure 14:**
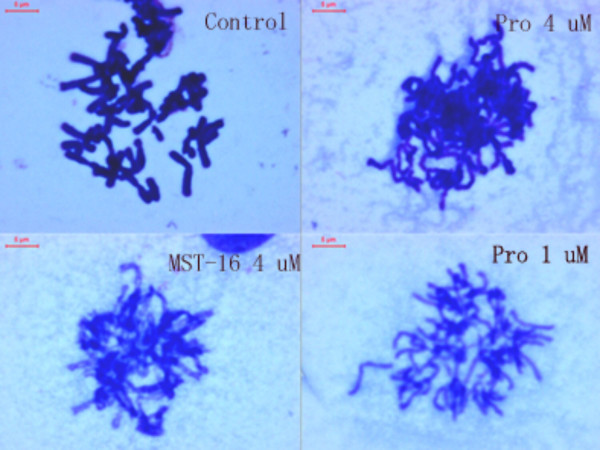
Chromosomal behaviors of human mammary tumor cell line- MDA-MB-468 incubated with bisdioxopiperazines. A: control; B: Probimane 4 μM; C: MST-16 4μM; D: Probimane 1μM.

Chromosome linkages, aggregations and segregation in tumor cells were blocked by both Pro and MST-16. Figure 13 and 14 show linkages and segregation blockade of chromosomes in cells treated with Pro and MST-16 at 4 μM. Despite this, chromosomes began to separate with each other, and their morphology became slimmer at lower concentration 1μM in both human mammary tumors of MDA-MB-436 cells and MDA-MB-468 cell lines *in vitro*. This chromosome poisoning action of Pro, MST-16 and ICRF-187 was seen at 1- 4 μM. In vehicle control group, chromosomes of tumor cells separated from each other very well. Although we only show typically one or two cells, the chromosomal characteristics in each group have an overall consistency (> 80 %) in each piece of preparation from cell treated with Pro, MST-16 and ICRF-187. They are common characteristics and phenotypes induced by the three compounds. In addition, there seems no difference in overall chromosome effects of Pro and MST-16 at equivalent concentrations (Figures 13 and 14), suggests that Pro and MST-16 act equally in this pathway.

## Discussion

**Figure 15:**
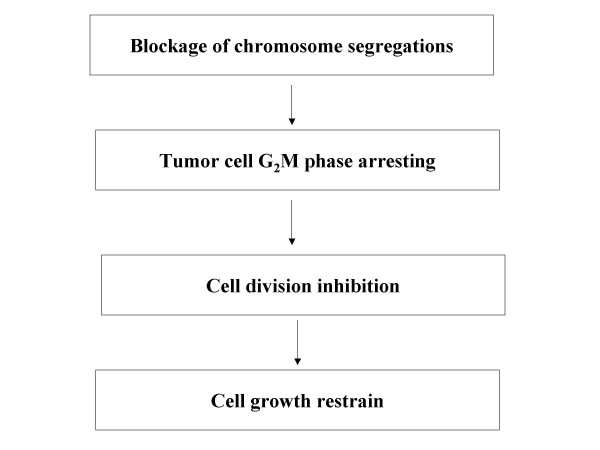
Proposed mechanism of anticancer effects for bisdioxopiperations.

Increased understanding over the mechanisms of bisdioxopopiperazines can greatly improve their indications and narrow down contraindicates in clinical practice. The explanations for the anticancer actions of bisdioxopiperazine are currently focusing on anti-angiogeneses [19,20] and tumor cell DNA alterations caused by topoisomerase II. Generally speaking, most angiogenesis inhibitors often have low cytotoxicity and are ineffective against larger tumor masses, and are better combined with cytotoxic drugs clinically [21,22]. This work on the anticancer activity of Pro and MST-16 shows that they act through the blocking of chromosomal segregation and G_2_/M phase arrests, causing complete inhibition of tumor cell division. Pro, MST-16 and ICRF-187 play similar roles at equi-molar concentrations. This pathway may be related to topoismerase II inhibition [23] as a possible mode of tumor growth inhibition, but is not suggested as a systematic approach through a cascade series. Two findings in this study need further discussion; (i) the effective ranges of Pro and MST-16 in the blocking of chromosome segregation, and causing G_2_/M phase arrests are 1- 4 μM, similar for Pro and MST-16. This suggests the two processes operate in the same course or cascade, and most possibly are directly linked; (ii) cyto-toxicity test (MTT) showed that Pro was more effective than MST-16. Lacking parallels in the effective dose ranges of Pro and MST-16 between cyto-toxicities and chromosome segregation – induced tumor inhibition can be explained by the fact that these effects of Pro and MST-16 do not strictly follow the same pathway given in Figure 15. Stronger cytotoxic effects of Pro against many other human tumor cell lines than original bisdioxopiperazines derivatives, especially on solid tumors, suggest some as yet undiscovered mechanism that Pro may have, and Pro may have better applications and require fewer drug combinations in the future.

This work shows that anticancer activities of Pro against lung cancer and leukemia are relatively greater than against other tumor typies. Cytotoxic and antimetastatic activities of Pro against lung tumor models *in vivo *have also been found [24]. Lung cancer is the most prevalent among all cancer categories, and is one of the deadliest cancers in the clinics. Targeted at lung cancers, Pro may offer better medical and economic benefits in the future.

For clinical chemotherapy, the paramount task is the balancing between treatment outcome and risks [25]. To optimize chemotherapeutic protocols containing bisdioxopiperazines, knowledge of its pharmacological parameters in terms of concentration- and time- responses are prerequisites. We found that Pro and MST-16 might act and accumulate longer in tumor cells than most of anticancer drugs. The peak of cytotoxicity of both Pro and MST-16 is on day 3, and not usually on day 2. This result and our early work of auto-radiography that Pro [26] persists longer in tumor tissues suggest that longer intervals may be used between treatments and less nursing responsibilities may arise, while maintaining high levels of tumor growth inhibitions. The long- term cytotoxic effects of Pro and MST-16 are more obvious in high metastatic tumor cell lines, which can explain the selective effects of compounds to tumor metastases. Early reports suggest that MST-16 needs to transform into ICRF-154 to exhibit its anticancer effects [27]. This work proves that MST-16 does not degraded to ICRF-154, and has a lower cytotoxic effects against tumor cells than Pro. Yet MST-16 can maintain a high activity in the cascade of the proposed mechanism – chromosome segregation blockage and cellular G_2_/M phase arrest, leading to inhibition of cell division (Figure 15). It further suggests this mechanism is not a pivotal pathway for cytotoxic activity against tumors.

## Conclusion

We suggest that Pro has a wider spectrum of cytotoxic effects against human tumor cells than other bisdioxopiperazines, especially on solid tumors. The cytotoxic pathway of Pro and MST-16 appears to be through chromosome segregation blocking and G_2_/ M phase arrests. Pro may be more potent than MST-16. High dose- and time- related responses of Pro than VCR, 5-Fu and Dox are seen that suggest a selectivity by Pro against tumor growth. It suggests that the family of bisdioxopiperazines may sustain their cytotoxic effects longer than other anticancer drugs.

## Methods

Pro and MST-16 were synthesized in this institute. Other chemical agents were purchased from sources stated below. The tumor cell lines were obtained from various sources and serially passaged in this lab.

### MTT method

The cells were maintained in RPMI 1640 (Gibco, Invitrogen Corporation, NY, USA) medium supplemented with 10 % FCS, streptomycin (100 μg/ml) and penicillin (100 units/ml). A density of 10^5 ^tumor cells /ml (90 μl) were seeded in 96-well plates for 24 h. Pro or MST-16 (10 μl), final concentrations indicated below, were added to each well for incubating for the next 48 h. 3-(4,5-dimethylthiazol-2-yl)-2,5-diphenyl tetrazolium bromide (MTT) (Sigma Company, USA) (5 mg/ ml, 20 μl) was added to each well. Four h later, 50 μl compound solution (10 % SDS- 5 % isobutyl alcohol-1 N HCl) were added and incubated under 5 % CO_2 _atmospheric condition for 24 h. Optical density at 570 nm was measured with a tunable microplate reader, VERSAmax, USA, each group was in triplicate samples and Pro or MST-16 were divided into 5 concentrations.

### Cell cycle analysis by cytometry

Tumor cells in exponential phase were exposed to Pro or MST-16. After 6 -24 h, cells were collected (300 × g, 10 min) and incubated with ice-cold PBS. Then fixed with ethanol and collected and washed with PBS by centrifugation (300 × g, 10 min). Cell deposition was added with PBS 1 ml and RNAse (5 μl) at 37°C bath for 15 min. Cells were dyed with 5 μl PI (2 mg/ml) in dark. Cells were measured for their DNA content by cytometry (Becton/Dickinson – FACS Calibur) after passing through a cell filter.

### Chromosome preparation protocols

Cell chromosome preparation was by a routine procedure. Human mammary tumor cells (MDA-MB-435 and MDA-MB-468) were seeded into a 6-well plate and maintained under an atmosphere of 5 % CO_2 _condition. When tumor cells covered about 60- 70 % of the surface, bisdioxopiperazines were added. Drug – treated cells were treated with hypotonic KCl, 0.075 M at 37°C for 30 min. Cell nuclei were fixed with fresh-prepared fixative solution [methanol/acetic acid, 3:1] for 5 min. Cell nuclei were collected by centrifugation (900 × g 15 min) and washed with fixative solution by centrifugation (1500 × g 20 min). Cell nuclei were dropped onto a cooled glass plate and placing overnight under a dehydrogenated atmosphere. The scattered chromosomes were dyed with a Giemsa solution for 15- 20 min and washed with tap water. Chromosomal behaviors were viewed and photographed by microscopy with an oil-lense (LEICA, Qwin image processing analysis system, Germany).

### Statistics

IC_50 _of agents were calculated by software in this lab and X ± SD was calculated from data of two groups.

## List of abbreviations used are

ADR (Dox), adriamycin; VCR, vincristine; 5-Fu, 5-fluorouracil; CaM, calmodulin; LPO, lipoperoxidation; Raz (ICRF-159 or ICRF-187), razoxane; Pro, probimane; PI, propidium iodide; MTT, 3-(4,5-dimethylthiazol-2-yl)- 2,5- diphenyl tetrazolium bromide;

## Authors' contributions

The work was designed by Da-Yong Lu and Jian Ding.

The manuscript was written by Da-Yong Lu.

Cytotoxic effects of compounds against human tumors was evaluated by Da-Yong Lu, Min Huang, Chen- Hui Xu, Wei- Yi Yang, Mei- Hong Li.

Cell cycle phase determination and plotting were completed by Da-Yong Lu and Lin- Jiang Tong.

Chromosome morphology was prepared and observed by Da-Yong Lu and Chao- Xin Hu.

The project was partly administered by Li Ping Lin and Xiong Wen Zhang.

Anticancer bisdioxopiperazines were provided by Wei Lu.
